# Transoral robotic surgery using the da Vinci single-port system: current evidence and clinical indications – a systematic review and meta-analysis

**DOI:** 10.1007/s11701-026-03540-0

**Published:** 2026-06-12

**Authors:** Tobias Niederegger, Emre Karakas, Murtaja Satea, Cosima C. Hoch, Gabriel Hundeshagen, Leonard Knoedler, Marc-Christian Metzger, Jonas Wüster

**Affiliations:** 1 Medical Faculty, University of Heidelberg, Heidelberg, Germany; 2 Department of Surgery, College of Medicine, University of Warith Al-Anbiyaa, Karbala, 56001 Iraq; 3 Division of Plastic Surgery, Microsurgery and Regenerative Medicine Laboratory, University of Wisconsin School of Medicine and Public Health, 600 Highland Avenue, Madison, WI 53705 USA; 4 Department of Otolaryngology, Head and Neck Surgery, TUM School of Medicine and Health, Technical University of Munich (TUM), Munich, Germany; 5 Department of Hand, Plastic and Reconstructive Surgery, Burn Trauma Center, BG Trauma Center Ludwigshafen, University of Heidelberg, Ludwig-Guttmann-Str. 13, 67071 Ludwigshafen, Germany; 6 Department of Oral and Maxillofacial Surgery, Charité – Universitätsmedizin Berlin, Corporate Member of Freie Universität Berlin and Humboldt- Universität zu Berlin, Augustenburger Platz 1, 13353 Berlin, Germany; 7 Department of Oral and Maxillofacial Surgery, Faculty of Medicine, University Medical Center Freiburg, Albert Ludwig University of Freiburg, Hugstetter Straße 55, 79106 Freiburg, Baden- Württemberg Germany

**Keywords:** Transoral robotic surgery, DaVinci, TORS, Single-port, Head and neck, Perioperative outcomes

## Abstract

The DaVinci Single-Port (SP) transoral robotic surgery (TORS) platform was developed to overcome spatial constraints and instrument collisions encountered with multiport systems in head and neck surgery. We systematically reviewed and meta-analyzed perioperative performance, safety, functional recovery, and oncologic outcomes of SP TORS. Following PRISMA 2020, we searched PubMed/MEDLINE, Embase, Cochrane Library, Web of Science, and the first 25 pages of Google Scholar through January 1, 2026. Peer-reviewed English-language clinical and cadaveric studies evaluating SP TORS were included. Three reviewers screened studies and extracted data. Random-effects meta-analyses were performed where appropriate. Fourteen studies (2019–2025) including 479 patients met inclusion criteria. Patient-level evidence was concentrated in oncologic cohorts, with additional smaller clinical applications in obstructive sleep apnoea-related tongue-base surgery (37 patients), hypopharyngeal foreign body removal (1 patient), parapharyngeal space surgery (2 patients), and SP-assisted reconstruction (3 patients), as well as preclinical feasibility studies. No study reported intraoperative conversion to multiport or open surgery. Pooled estimates were console time 60.04 min (95% CI 29.56–90.51; I² = 99.1%), docking time 10.13 min (0.47–19.79; I² = 97.9%), operative time 57.72 min (37.23–78.21; I² = 0.0%), length of stay 10.63 days (2.43–18.82; I² = 99.8%), and blood loss 24.16 mL (− 6.95 to 55.27; I² = 86.8%). Bleeding was the most consistently reported surgical complication, with major bleeding requiring operative management reported in larger cohorts but remaining uncommon. Functional recovery suggested early oral intake, although timing was inconsistently reported. Margin status was predominantly R0 in several oncologic cohorts, although one matched cohort reported a lower R0 rate. SP TORS appears feasible across indications, but current evidence is limited by nonrandomized designs, inconsistent long-term reporting, and between-study heterogeneity.

**Supplementary Information:**

The online version contains supplementary material available at 10.1007/s11701-026-03540-0.

## Introduction

Transoral robotic surgery (TORS) has transformed the landscape of minimally invasive head and neck surgery by enabling precise access to anatomically constrained regions of the oropharynx, larynx, and hypopharynx while reducing surgical morbidity associated with traditional open approaches. Since its introduction in 2009, TORS has been propelled by continuous technological refinement, shifting from early multi-port platforms to more compact, ergonomic solutions that address the spatial and visual limitations inherent to transoral work [1–3]. Multi-port systems laid the foundation for robotic transoral procedures by offering three-dimensional visualization and improved instrument articulation. However, their bulk, external arm collisions, and narrow working corridors guided the development of the next-generation single-port system, the DaVinci Single-Port [4].

The Single-Port (SP) robotic platform represents a conceptual evolution toward more streamlined, anatomically compatible technology. By consolidating the camera and instruments into a single cannula, the SP system provides enhanced access to deep, curved anatomical spaces, improved visualization angles, and better instrument triangulation within confined transoral cavities [5, 6]. Clinically, single-port TORS (SP-TORS) promises reduced operative trauma, fewer docking constraints, and more intuitive manipulation, factors that may translate into shorter operative times, less postoperative pain, and quicker functional recovery. Despite these theoretical and early clinical advantages, debate persists over whether the SP system truly surpasses established multi-port platforms with respect to oncological, functional, and perioperative outcomes [7]. The relative benefits of enhanced access must be weighed against new limitations, such as reduced instrument independence, learning-curve demands, and variable cost-effectiveness [8–10].

As the field continues to expand and institutions adopt the SP technology at an accelerating pace, a comprehensive synthesis of the current evidence is essential. Existing studies remain heterogeneous in design, indications, and outcome reporting, and no unified framework has been established to evaluate the clinical performance and safety profile of SP-TORS. Therefore, the aim of this systematic review was to summarize and critically assess the available evidence on the DaVinci SP in TORS, spanning operative metrics, functional recovery, complication profiles, and oncologic outcomes. By consolidating current data, this review seeks to clarify the clinical role of SP technology, identify gaps in the literature, and inform future surgical innovation and comparative research in TORS.

## Methods

This systematic review and meta-analysis was conducted in accordance with the PRISMA 2020 reporting standards to promote methodological transparency and completeness [11]. The review protocol was registered in advance with PROSPERO (CRD420251270906).

### Systematic search

A comprehensive literature search was conducted across multiple databases, PubMed/MEDLINE, EMBASE, the Cochrane Library, Web of Science, and the first 25 pages of Google Scholar, to identify all relevant studies published up to January 1st, 2026. The search strategy was structured around two core concepts: “transoral robotic surgery (TORS)” and “single-port (SP) robotic systems,” which were combined using the Boolean operator AND. Synonyms, related keywords, and MeSH terms were incorporated to maximize sensitivity. For Google Scholar, the first 25 result pages were screened using the predefined search string. This threshold was selected a priori to balance sensitivity and reproducibility, as Google Scholar ranks results by relevance and later pages commonly yield increasingly non-specific records. Detailed database-specific search strategies are provided in Supplementary Digital Content 1. Additional articles were located through manual screening of reference lists from included studies.

Studies were eligible if they evaluated the clinical use, technical performance, surgical outcomes, feasibility, or anatomical applicability of the DaVinci SP system in TORS, including human clinical studies and cadaveric investigations. Only peer-reviewed, full-text articles published in English were included, irrespective of study design. Animal studies, non–peer-reviewed studies, conference abstracts, narrative reviews, systematic reviews and studies unrelated to transoral robotic applications were excluded.

**Fig. 1:**
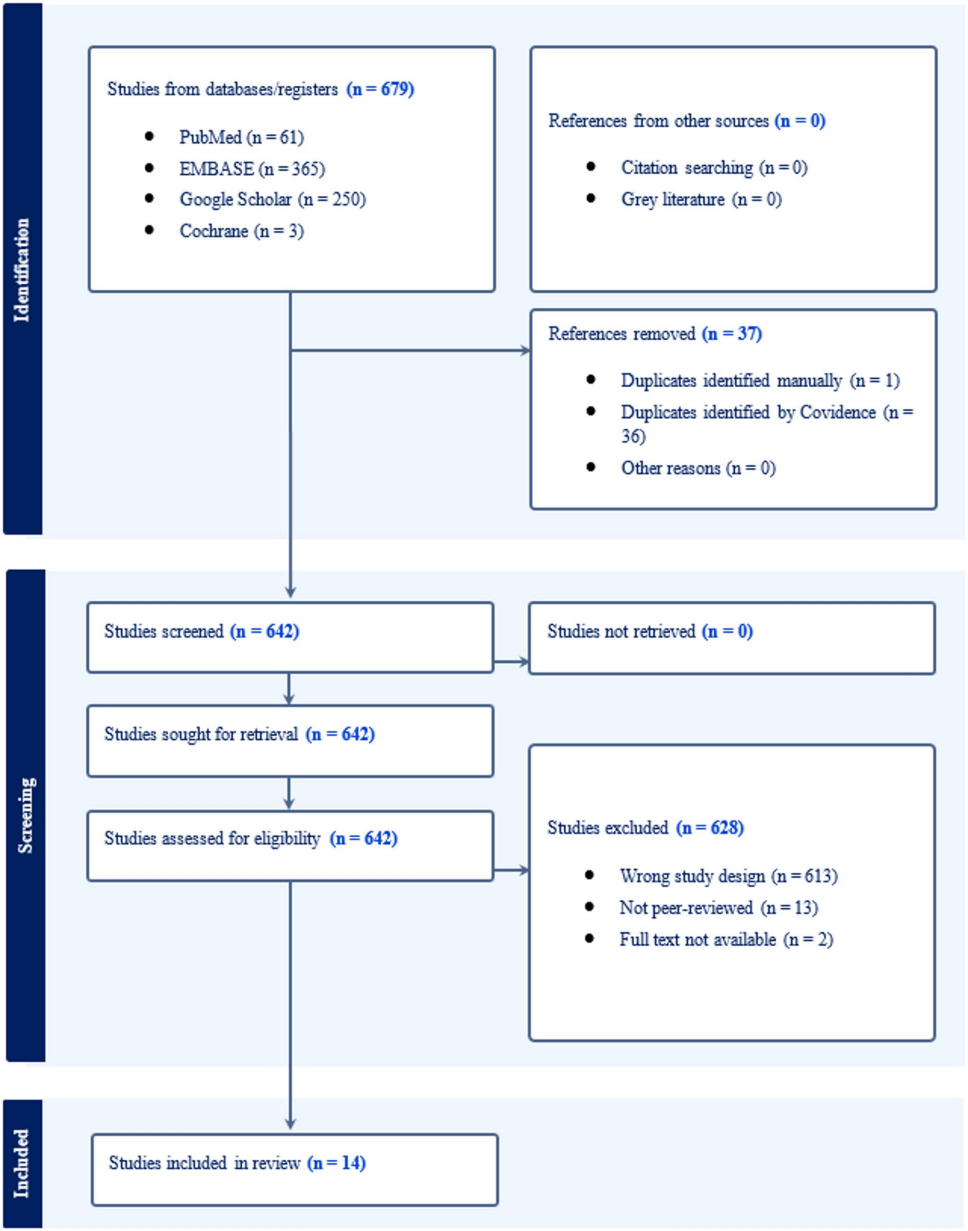
PRISMA 2020 flowchart

Three reviewers independently screened titles and abstracts, followed by full-text assessment to determine eligibility. Discrepancies were resolved by consensus in consultation with a senior reviewer. The study selection process is summarized in the PRISMA 2020 flow diagram (Fig. 1).

### Quality assessment

The methodological quality of the included studies was evaluated using established appraisal tools selected according to study design. Clinical studies were assessed with the Newcastle–Ottawa Scale (NOS), which examines methodological rigor across three domains: cohort selection, comparability of study groups, and adequacy of outcome measurement. The NOS assigns a maximum of nine points, with higher scores indicating higher study quality, and is widely used in observational research, including retrospective cohort studies, prospective clinical series, and case–control analyses [12]. Case reports and case series were assessed using the Joanna Briggs Institute (JBI) critical appraisal checklists for case reports and case series, respectively, to allow a design-appropriate evaluation of descriptive clinical evidence [13]. In addition, the broader strength of the evidence was categorized according to the Oxford Centre for Evidence-Based Medicine (OCEBM) Levels of Evidence (LOE), which stratify research from Level I (well-designed randomized controlled trials) to Level V (foundational evidence) based on study design and methodological rigor [14]. A detailed summary of all quality assessment results is provided in Supplementary Digital Content 2, 3 and 4.

### Data extraction

Data from each included study were collected using a standardized extraction framework. Bibliographic information (DOI, first author, title, year, and region) and study characteristics (design, intervention type, robotic platform) were recorded. Patient data included sample size, age, sex, and tumor-specific factors such as site, stage, and indication (oncologic vs. benign). Operative variables extracted comprised operative and docking times (mean/median with SD or IQR), need for intraoperative conversion to multiport or open surgery, and robotic system–related issues such as instrument collisions. For time-related outcomes, including docking time, console time, and operative time, definitions and terminology were extracted according to the original study authors’ definitions and were not retrospectively reclassified unless sufficient detail supported equivalence. Surgical and oncologic outcomes included margin status (R0 and margin width when available), postoperative complications, and oncologic endpoints (locoregional control, survival, adjuvant treatment). Complication data were extracted according to the classification system used in each primary study. When Clavien–Dindo grading was reported, the original grades were retained; grade III events were interpreted as complications requiring surgical, endoscopic, or radiologic intervention, and grade IV events as life-threatening complications requiring intensive-care-level management. Functional recovery metrics, time to oral intake, swallowing and voice outcomes, and length of hospital stay, were also documented. Each study’s main conclusions regarding the feasibility, performance, and clinical value of single-port TORS were summarized.

### Statistical analysis

Random-effects meta-analyses were performed to obtain pooled estimates of key numerical outcome data because clinical and methodological heterogeneity was expected across indications, study designs, and outcome definitions. Between-study variance was estimated using restricted maximum likelihood (REML), and confidence intervals were calculated using the Hartung–Knapp adjustment to provide more conservative inference in the setting of small numbers of heterogeneous studies [15]. Heterogeneity was assessed using Cochran’s *Q* and I² statistics. Outcomes were analyzed separately according to the terminology used by the original study authors, particularly for docking time, console time, operative time, and length of stay, and differently defined perioperative time variables were not retrospectively merged unless the reported definitions supported comparability. For numerical outcomes reported as means without standard deviations, but with available ranges, the standard deviation was approximated using the formula SD ≈ (maximum − minimum)/4 where necessary for quantitative synthesis. Conversion of medians or interquartile ranges to means and standard deviations was not performed. Where applicable, subgroup comparisons were assessed using the Q-test for between-group heterogeneity, with heterogeneity reported using I². Study-level and pooled effect estimates are reported with 95% confidence intervals, and forest plots were generated to visualize individual and pooled results. Given the non-uniform definitions and reporting across studies, pooled estimates were interpreted as exploratory descriptive summaries rather than definitive quantitative benchmarks. All analyses were conducted in R version 4.5.1, and a p-value < 0.05 was considered significant.

## Results

**Fig. 2:**
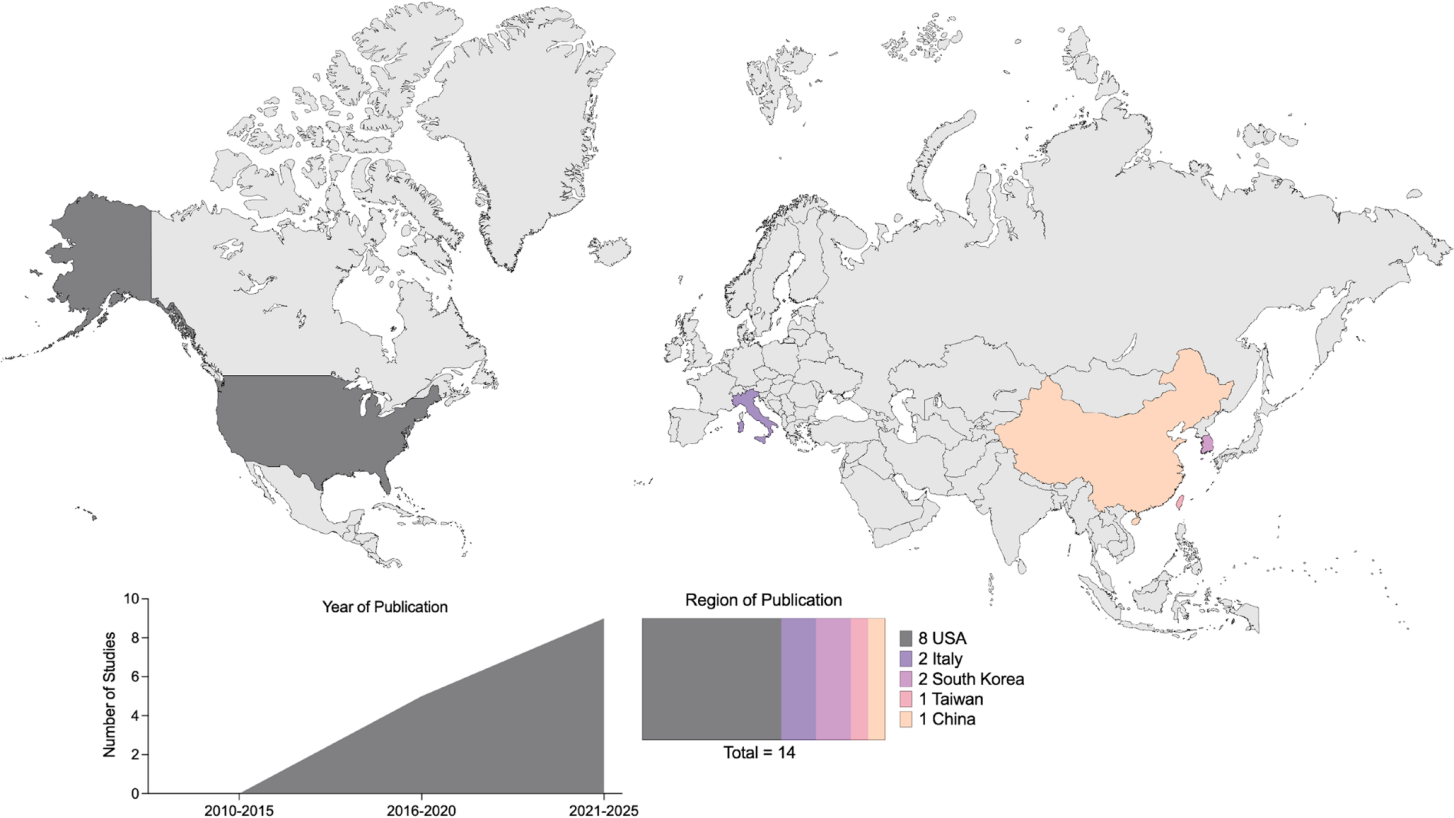
Demographic and timeline distribution of studies

The systematic search yielded *n* = 679 studies. After screening with the prespecified inclusion and exclusion criteria, 14 studies were included in the systematic review [6, 9, 16–27]. Year of publication ranged from 2019 to 2025. The majority of studies were conducted in the United States (*n* = 8, 57%), followed by Italy (*n* = 2, 14%), South Korea (*n* = 2, 14%), Taiwan (*n* = 1, 7.1%) and China (*n* = 1, 7.1%) (Fig. 2). A total of *n* = 479 patients were analyzed. The mean (SD) patient age was 57.4 (12.2) years. The patient cohort was predominantly male (*n* = 274, 57% vs. *n* = 57, 12% female) and unknown in *n* = 148 (31%).

Study designs included *n* = 3 (21%) phase II controlled clinical trials, *n* = 4 (29%) cohort studies, *n* = 3 (21%) case series, *n* = 2 (14%) case reports and *n* = 2 (14%) preclinical cadaver feasibility studies. The mean (SD) NOS score was 5.0 (1.0), and the LOE ranged from Level 2 to Level 5 (foundational evidence), indicating an overall moderate methodological quality (Supplementary Digital Content 2 and 3). Full study demographics are provided in Table 1.

**Table 1:** Study demographics

DOI	First author et al.	Title	Year of Publication	Region of publication	Study type	Sample size	Patient age	Patient sex	Intervention type	Robotic type
10.1245/s10434-019-07802-0	Park et al.	The First Human Trial of Transoral Robotic Surgery Using a Single-Port Robotic System in the Treatment of Laryngopharyngeal Cancer	2019	South Korea	Retrospective case series	41	Mean 60 years (range 41–78)	35 male (85.3%), 6 female (14.7%)	TORS- upper aerodigestive tract malignancy (oropharynx/larynx/hypopharynx): lateral oropharyngectomy, tongue base resection, hypopharyngectomy	Da Vinci SP single-port robotic system
10.1001/jamaoto.2019.2654	Holsinger et al.	A Next-Generation Single-Port Robotic Surgical System for Transoral Robotic Surgery: Results From Prospective Nonrandomized Clinical Trials	2019	USA	Phase 2, multicenter, prospective non-randomized clinical trial	47	Mean 61.0 ± 8.3 years	39 male (83%), 8 female (17%)	Single-port flexible TORS-oropharynx: staging/endoscopy (benign) and TORS resection for malignant T1-T2 tumors	Next-gen da Vinci SP (Single Port Surgical System)
10.1002/hed.25676	Orosco et al.	Transoral supraglottic laryngectomy using a next-generation single-port robotic surgical system	2019	USA	Preclinical cadaver feasibility/anatomic study	1 human cadaver	N/A	N/A	Single-port flexible robotic supraglottic laryngectomy (cadaver): fresh-frozen cadavers; en bloc supraglottic resections	Da Vinci SP single-port flexible robotic system
10.1016/j.oraloncology.2019.05.018	Chan et al.	Prospective clinical trial to evaluate safety and feasibility of using a single-port flexible robotic system for transoral head and neck surgery	2019	China	Prospective Phase II, IDEAL stage 2, single-arm clinical trial of safety and feasibility	21	Median 60 years (range 41–75)	15 male (71.4%), 6 female (28.6%)	Single-port flexible TORS-head and neck benign/malignant (naso-/oro-/larynx/hypopharynx): oropharyngectomy, TBR, partial laryngectomy, local excisions	Da Vinci SP single-port robotic system
10.1002/hed.26143	Van Abel et al.	One-year outcomes for da Vinci single-port robot for transoral robotic surgery	2020	USA	Retrospective cohort with historical Si comparison	78	Mean age 61.9 ± 9.1 years	Sex: 41 male (87.2%), 6 female (12.7%)	Oropharyngeal tumors (mostly HPV+ OPSCC): SP cases pathology (reviewed *n* = 47): OPSCC 41/47; other entities 1/47 each (adenoCA, MEC, lymphoma, PLGA, benign/necrotic tonsil)	Da Vinci SP vs. da Vinci Si (legacy multiport)
10.1002/hed.26794	Mendelsohn et al.	Single-port transoral robotic surgery hypopharyngectomy	2021	USA	Case report	1	82	M	Single-port TORS hypopharyngectomy: resectable hypopharyngeal squamous cell carcinoma -medial pyriform sinus wall	Da Vinci SP single-port robotic system
10.1002/ohn.287	Sampieri et al.	Single-Port Versus Multiport da Vinci System for Transoral Robotic Surgery of Hypopharyngeal and Laryngeal Carcinoma	2023	Italy	Retrospective cohort study with propensity score–matched comparison of SP vs. multiport (Si/Xi) TORS	112	Mean age overall: 66.51 ± 8.32 years	N/A	TORS for larynx/hypopharynx SCC: supraglottic partial laryngectomy and partial hypopharyngectomy; da Vinci SP vs. multiport Si/Xi	Da Vinci SP vs. multiport (Si/Xi) comparison
10.1002/rcs.2510	San Juan et al.	Evaluation and application of CO2 laser fiber delivery for single port transoral robotic surgery	2023	USA	Preclinical feasibility and usability study in a laboratory setting using human cadaver specimens and trainee surveys	17 residents	N/A	N/A	Cadaveric oropharyngeal dissection (da Vinci SP): SP and monopolar electrocautery vs. SP and CO₂ laser (flex fiber and drop-in guide) and handheld CO₂ exercise on cadaveric liver	Da Vinci SP and drop-in guide and FiberLase CO₂ fiber; comparators: SP electrocautery and handheld CO₂
10.1016/j.oraloncology.2023.106629	Costantino et al.	Transoral robotic surgery in oropharyngeal squamous cell carcinoma: A comparative study between da Vinci Single-Port and da Vinci Xi systems	2024	Italy	Single-center retrospective cohort study comparing da Vinci SP vs. Xi in OPSCC patients treated with TORS after neoadjuvant chemotherapy	209 patients total (SP *n* = 136, Xi *n* = 73)	SP 59.1 ± 9.6	SP 80% male	TORS for oropharyngeal squamous cell carcinoma post-neoadjuvant chemo: standardized neoadjuvant chemotherapy and simultaneous neck dissection and prophylactic facial/lingual artery ligation	Da Vinci SP vs. da Vinci Xi (multiport)
10.1002/lary.30882	Stefan et al.	Single-Port Robotic Removal of a Submucosal Foreign Body in the Distal Hypopharynx	2024	USA	Case report	1	55	F	Single-port transoral foreign body removal: hypopharyngeal anterior cervical discectomy and fusion screw after failed transcervical attempts	Da Vinci SP single-port robotic system
10.1016/j.oor.2024.100547	Gorelik et al.	Single-port robotic surgery for pleomorphic adenomas of the parapharyngeal space	2024	USA	Case series	2	62 and 39	F	Da Vinci SP transoral parapharyngeal space tumor resection (benign): transoral corridor; palatal incision: superior constrictor; extracapsular dissection	Da Vinci SP single-port robotic system
10.1007/s11701-025-02699-2	Fang et al.	Evaluation of single-port TORS tongue base resection for obstructive sleep apnea: safety and patient outcomes	2025	Taiwan	Non-randomized, single-arm phase II observational trial	12	Mean age 40.3 ± 10.1 years	12 male (100%), 0 female	TORS tongue base resection/lingual tonsillectomy for OSA: single-level or multilevel oropharyngeal surgery	Da Vinci SP single-port robotic system
10.1002/hed.28007	Jeong et al.	First Prospective Study on Single-Port Robotic Tongue Base Resection for Sleep Apnea	2025	South Korea	Prospective, single-center, single-surgeon clinical investigation	25	Mean 37.9 ± 11.9 years	22 male (88%), 3 female (12%)	Single-port TORS tongue base resection for OSA (multilevel): expansion sphincter pharyngoplasty ± septoplasty/turbinoplasty	Da Vinci SP single-port robotic system
10.1007/978-3-031-96837-2_3	Chen et al.	Single-Port Robotic Free Flap Inset Following Transoral Robotic Surgery	2025	USA	Case series	3	Mean age 61.3 years	F	Da Vinci SP TORS for oropharyngeal cancer and reconstruction: tumor resection followed by robotic free flap inset (radial forearm free flap or anterolateral thigh flap) on same SP platform	Da Vinci SP used for TORS resection and robotic free-flap inset (cobra scope; 3 × 6-mm articulating instruments

### Application of single-port transoral robotic surgery across various intervention types

The included studies evaluated SP-TORS across oncologic, non-oncologic, reconstructive, and preclinical settings. Patient-level evidence was concentrated in oncologic cohorts, including laryngo-pharyngeal and upper aerodigestive tract malignancies in Park et al. (41 patients), malignant oropharyngeal resections within the Holsinger et al. prospective cohort (40 malignant cases among 47 enrolled patients), mixed benign and malignant head and neck procedures in Chan et al. (8 malignant cases among 21 patients), oropharyngeal squamous cell carcinoma in Van Abel et al., hypopharyngeal and laryngeal carcinoma in Sampieri et al. (112 patients), and oropharyngeal squamous cell carcinoma treated after neoadjuvant chemotherapy in Costantino et al. (136 SP patients within a 209-patient SP-versus-Xi cohort) [9, 16–18, 20, 22].

Smaller non-oncologic or adjunctive clinical applications included obstructive sleep apnoea-related tongue-base surgery in 37 patients across two cohorts (Fang et al., *n* = 12; Jeong et al., *n* = 25), transoral removal of a hypopharyngeal foreign body in 1 patient, parapharyngeal pleomorphic adenoma resection in 2 patients, and SP-assisted free-flap inset after oropharyngeal resection in 3 patients [25, 26]. Preclinical feasibility work consisted of cadaveric supraglottic laryngectomy and oropharyngeal dissections comparing monopolar electrocautery with CO₂ laser delivery via a drop-in guide system. Overall, the current patient-level evidence remains primarily concentrated in oncologic applications, while non-oncologic, reconstructive, and preclinical uses are supported by smaller early reports (Table 1).

### Operative efficiency and system performance metrics

Operative efficiency outcomes were heterogeneously reported and are summarized in Table 2. Across comparative studies, SP-TORS generally demonstrated shorter setup or docking times than multiport systems. Park et al. reported shorter setup time with the SP system than with the da Vinci Si system, while Sampieri et al. and Costantino et al. reported shorter docking times for SP-TORS compared with multiport platforms [16, 20, 22]. Console and operative times were more variable and appeared to depend on procedure type, subsite, exposure, and institutional workflow.

**Table 2:** Overview of surgical details

DOI	First author et al.	Title	Tumor characteristics	Operative metrics	Intraoperative conversion	Surgical margin status	Complications	Functional outcomes	Oncological outcomes	Robotic system metrics
10.1245/s10434-019-07802-0	Park et al.	The First Human Trial of Transoral Robotic Surgery Using a Single-Port Robotic System in the Treatment of Laryngopharyngeal Cancer	Malignant head and neck cancers: Primary subsites: tonsil 14 (34.1%), base of tongue 10 (24.4%), pyriform sinus 5 (12.2%), false vocal cord 4 (9.6%), posterior pharyngeal wall 2 (4.8%), true vocal cord 2 (4.8%), aryepiglottic fold 1 (2.4%), other 3 (7.7%)	Setup/operation time (single port vs. da Vinci Si): Setup 8.04 ± 4.62 min and operation 56.95 ± 30.0 min (single port) vs. setup 13.04 ± 8.91 min and operation 107 ± 44 min (da Vinci Si)	None	Final margins by site: Laryngeal cancer: negative 5 (71%), positive 2 (29%); hypopharyngeal cancer: negative 5 (71%), positive 2 (29%); oral cavity/oropharyngeal cancer: negative 20 (91%), positive 2 (9%)	Bleeding and prevention: Mean blood loss 126.5 mL; no transfusions. One postoperative tonsillar bleed treated with bipolar cautery under general anaesthesia. Prophylactic neck ligation of the lingual artery or superior laryngeal artery used for tongue base resection/hypopharyngectomy to reduce bleeding	Airway, feeding, and length of stay: Oropharyngeal cases used nasotracheal intubation with no airway obstruction from oedema/bleeding. Hypopharyngeal cancer cases had temporary tracheostomy with decannulation in ≤ 28 days, oral intake started in ≤ 11 days, and mean hospital stay 23.3 days	Adjuvant therapy by cancer site: Laryngeal cancer (*n* = 7): concurrent chemoradiotherapy 3 (43%), radiotherapy 1 (14%), surgery alone 3 (43%). Hypopharyngeal cancer (*n* = 7): concurrent chemoradiotherapy 6 (86%), surgery alone 1 (14%). Oral cavity/oropharyngeal cancer (*n* = 22): concurrent chemoradiotherapy 13 (59%), radiotherapy 4 (18%), surgery alone 5 (23%)	Setup time and collision notes: Setup time 8.04 ± 4.62 min (da Vinci Single Port). Authors state earlier multiport systems had more arm collisions in the narrow pharynx, whereas the single-port arm “joggle joints” increase freedom of movement and help minimize collisions
10.1001/jamaoto.2019.2654	Holsingeret al.	A Next-Generation Single-Port Robotic Surgical System for Transoral Robotic Surgery: Results From Prospective Nonrandomized Clinical Trials	Oropharyngeal tumors (*n* = 47): 40 malignant, 7 benign/indeterminate. Subsites: tonsil 23 (49%), tongue base 16 (34%), glossopharyngeal sulcus 6 (13%), soft palate 2 (4%). Histology: 38 squamous cell carcinomas, 2 minor salivary gland carcinomas	Estimated blood loss: Mean 15.4 ± 23.9 mL; no transfusions	None	Cancer cases: Negative margins in 39/40 (98%); 1/40 (2%) had microscopic tumor at final histology	Adverse events: 2 patients (4%) had grade III–IV postoperative haemorrhage requiring return to the operating room. Additional events included grade 1 (taste disturbance, voice change from vocal cord haematoma, antibiotic allergy, swallowing difficulty/secretions, fall) and grade 2 (minor bleeds controlled in clinic, cough/pneumonia without admission, nasal regurgitation, trismus). Serious events included Clostridioides difficile enterocolitis with dehydration and delirium with pneumonia; no deaths	Early oral intake and swallowing measure: Within 30 days, 45/47 (96%) were eating by mouth without a percutaneous endoscopic gastrostomy tube. Mean length of stay at United States centers 4.2 ± 1.9 days; swallowing outcomes assessed with the MD Anderson Dysphagia Inventory preoperatively, 2 weeks postoperatively, and at final follow-up	N/A	Collision-avoidance features: Arm deployment can be offset to reduce proximal and distal instrument-tip collisions; a navigator panel is described as enabling arm positioning that minimizes collisions despite the extra arm
10.1002/hed.25676	Orosco et al.	Transoral supraglottic laryngectomy using a next-generation single-port robotic surgical system	N/A (cadaveric feasibility study; no tumors)	Instrumentation feasibility: Three instruments plus a flexible camera used with minimal collision and preserved arm range of motion	N/A	N/A	N/A	N/A	N/A	Cadaveric supraglottic laryngectomy feasibility: Three instruments plus a flexible camera were deployed with minimal collision and adequate access/visualization to perform transoral robotic supraglottic laryngectomy
10.1016/j.oraloncology.2019.05.018	Chan et al.	Prospective clinical trial to evaluate safety and feasibility of using a single-port flexible robotic system for transoral head and neck surgery	Mixed sites and pathology: Clinical sites: nasopharynx 1, oropharynx 15, larynx 4, hypopharynx 2; overall pathology 8 malignant and 13 benign/functional lesions. Malignant cases included human papillomavirus-positive and -negative squamous cell carcinomas (tonsil/tongue base) and supraglottic squamous cell carcinoma; benign/functional cases included obstructive sleep apnoea-related lingual tonsil hypertrophy, papillomas, strictures, and benign laryngeal lesions; several early tumors were described as T1-T2	Docking/operative time and blood loss: Docking 7 min (range 3–29); operative time 61.1 min (range 35–215); estimated blood loss 39.2 mL (range 5–100)	None	Selected malignant resections (reported subset): All listed oropharyngeal and laryngeal squamous cell carcinoma resections achieved negative margins (human papillomavirus–positive tonsil *n* = 5, tongue base *n* = 2, supraglottic larynx *n* = 2)	No serious adverse events and none attributed to the robot within 30 days. Complications in 8/21, Clavien–Dindo grade I (5), II (1), IIIa (2); examples included dental fracture, small tongue laceration from retractor, transient taste change, vocal cord haematoma from intubation, self-limited granulation bleeding, drug allergy, and mild tonsillectomy-site bleeding controlled at bedside	Length of stay and dysphagia/pain trajectories: Length of stay mean 10.4 days (range 1–30). In the oropharyngeal squamous cell carcinoma subset, MD Anderson Dysphagia Inventory scores worsened at 2 weeks and improved toward baseline by 30 days; pain (visual analogue scale) rose at 2 weeks (3.9 ± 3.1) then decreased by 30 days (1.6 ± 1.8)	N/A	System configuration and assistant access: Docking time mean 7 min (range 3–29). Described as a single cannula with stereoscopic camera and three 6 mm “snake-like” arms with an elbow joint enabling access to naso-/oro-/hypopharynx and larynx; collision rates not quantified. Assistant suction workspace was limited by the extra arm and mitigated with a nasally placed suction catheter
10.1002/hed.26143	Van Abel et al.	One-year outcomes for da Vinci single-port robot for transoral robotic surgery	Oropharyngeal squamous cell carcinoma comparison (single port *n* = 41 vs. multiport *n* = 163): Single port had more base-of-tongue primaries (56.1%) and fewer tonsil primaries (43.9%); human papillomavirus positivity 90.2%. Pathologic tumor stage distribution was similar (pT1 41.5%, pT2 41.5%, pT3 14.6%, pT4 2.4%), as was nodal stage (pN0 18.4%, pN1 68.4%, pN2 13.2%)	Console time (single port): Overall (available *n* = 71) mean 82 min (range 18–199). Learning curve in oropharyngeal squamous cell carcinoma: first 20 cases 96.3 ± 32.4 min vs. last *n* = 21 cases 79.1 ± 22.4 min	None	Chart-reviewed single-port cases 100% negative (*n* = 47). In the oropharyngeal squamous cell carcinoma comparison, single port 0% positive vs. multiport da Vinci Si 3.1% positive (*p* = 0.2)	Haemorrhage 4/78 (overall bleed rate 5.1%). Thirty-day mortality 2/78 (2.6%): one death after tonsillectomy haemorrhage and one from pulmonary embolism	Single port vs. da Vinci Si (oropharyngeal squamous cell carcinoma with unilateral neck dissection): Length of stay 2.1 ± 0.6 vs. 3.2 ± 1.4 days (*p* < 0.01). No significant differences in console time (87.9 ± 29.0 vs. 85.6 ± 40.5 min) or anaesthesia time (295 ± 92 vs. 315 ± 101 min); negative margins 100% vs. 92.6%	N/A	Single-port advantages and limitations (qualitative): Advantages included a single cannula with three 6 mm articulated arms, flexible “cobra” camera, integrated bipolar energy, navigator display, and occasional third-arm retraction. Challenges included internal arm collisions and “skipping” over the camera in base-of-tongue work, fixed focal length and relatively bulky camera, parallel-segment constraints, and a learning curve for camera/arm positioning and third-arm use
10.1002/hed.26794	Mendelsohn et al.	Single-port transoral robotic surgery hypopharyngectomy	Hypopharyngeal squamous cell carcinoma: Medial wall of the right pyriform sinus; stage T1N0M0	N/A	N/A	N/A	N/A	Voice and airway described as excellent; oral feeding started postoperative day 3; discharge postoperative day 4	Final pathology confirmed T1N0M0 with no adverse features; no adjuvant therapy indicated	Reach and third-arm retraction: Improved flexibility enabled access to more distal upper aerodigestive tract targets; the third arm was emphasized as a readily used distal retractor compared with multiport systems
10.1002/ohn.287	Sampieri et al.	Single-Port Versus Multiport da Vinci System for Transoral Robotic Surgery of Hypopharyngeal and Laryngeal Carcinoma	Laryngeal/hypopharyngeal squamous cell carcinoma (*n* = 185): Primary site: hypopharynx 56% (104/185), larynx 44% (81/185); subsites: pyriform sinus 41% (76), supraglottis 22% (41), glottis 22% (40). Clinical tumor stage: T1 25%, T2 27%, T3 42%, T4a 5.4%	Docking/console time (single port vs. multiport): Docking 6.45 ± 3.11 min vs. 8.84 ± 4.67 min; console 42.70 ± 13.72 min vs. 53.91 ± 29.38 min	None	Matched cohort (*n* = 112): Negative 59/112 (53%), positive 53/112 (47%). By platform, positive margins were 52% (multiport) vs. 43% (single port), not significant (*p* = 0.34)	Thirty-day complications (Clavien–Dindo > II): 4/112 (3.6%) overall. Single-port events included failed nasogastric tube placement due to laryngeal swelling requiring percutaneous endoscopic gastrostomy, and a pharyngeal fistula requiring revision with a pedicled flap	Tracheostomy, feeding, and hospital stay (single port vs. multiport): Tracheostomy performed in 85% overall; decannulation time 17.86 ± 10.93 days (single port) vs. 16.00 ± 10.03 (multiport; *p* = 0.046). Enteral feeding duration 14.96 ± 8.43 vs. 16.61 ± 9.02 days (not significant); hospital stay 21.55 ± 7.35 vs. 23.48 ± 11.22 days (not significant)	After treatment (upfront transoral robotic surgery or neoadjuvant chemotherapy followed by transoral robotic surgery), 25% were pathologic stage T3-T4a; similar between multiport (25%) and single port (23%, *p* = 0.32)	Exposure and mouth opening (matched comparison): Mean interincisor distance 36.67 ± 4.12 mm; exposure rated “fair/good” in 76%, similar between single port and multiport. Single-port improvements noted included a flexible “cobra” camera, 6 mm arms with two internal joints, reduced collisions, and the ability to use a third arm for retraction (not used with multiport)
10.1002/rcs.2510	San Juan et al.	Evaluation and application of CO2 laser fiber delivery for single-port transoral robotic surgery	N/A (cadaveric feasibility study; no tumors)	N/A	N/A	N/A	No adverse events or safety issues with the carbon dioxide laser fiber or robotic drop-in guide during 17 cadaveric dissections	N/A	N/A	Carbon dioxide laser integration and usability: Carbon dioxide laser delivered via a flexible FiberLase fiber held in a robotic drop-in guide; activation by foot pedal. System Usability Scale scores were similar overall, but single port plus carbon dioxide laser was rated more complex/cumbersome than handheld laser by low-experience trainees (*p* < 0.05); experienced users rated it comparable
10.1016/j.oraloncology.2023.106629	Costantino et al.	Transoral robotic surgery in oropharyngeal squamous cell carcinoma: A comparative study between da Vinci Single-Port and da Vinci Xi systems	Oropharyngeal squamous cell carcinoma only (*n* = 209): Subsites: tonsil 156 (75%), base of tongue 53 (25%). p16 positivity 154/209 (74%) (higher in single port 82% vs. multiport 59%). Pre-treatment clinical stage: T1 17%, T2 51%, T3 15%, T4a 17%; post-treatment pathologic stage: pT0 33%, pT1 38%, pT2 21%, pT3 5.3%, pT4a 3.3	Docking/console time and tracheotomy (single port vs. da Vinci Xi): Docking overall 7.1 ± 3.8 min; single port 5.7 ± 2.5 vs. Xi 10.0 ± 4.4 (*p* < 0.001). Console overall 80.2 ± 33.2 min; single port 76.3 ± 30.7 vs. Xi 88.1 ± 36.9 (*p* = 0.06). Intraoperative tracheotomy 88/209 (42%); single port 44% vs. Xi 38% (*p* = 0.42)	None	Single port margins: Negative 96 (71%), close 13 (9.6%), positive 27 (20%); mean margin distance in negative/close cases 3.1 ± 2.9 mm (no difference vs. da Vinci Si reported)	Major postoperative bleeding requiring haemostasis under general anaesthesia 6/209 (2.9%); single port 5 (3.7%) vs. multiport 2 (2.7%) (*p* = 0.90). Two deaths in the single-port group at 20 and 29 days were due to medical causes unrelated to surgery	Longer-term dependence and length of stay (single port vs. da Vinci Xi): Hospital stay 18.3 ± 4.7 days overall (no difference by platform). At 6 months, tracheostomy tube dependence 3/209 (1.6%) (all single port) and feeding tube dependence 1/209 (0.5%) (single port)	N/A	Collision phenomena and third-arm use (single port vs. multiport): Docking was shorter with single port (5.7 vs. 10.0 min). Internal collisions (“chopstick” phenomenon) were described as controllable and improved with experience and navigator guidance; external conflicts at the oral entry were reduced due to the single cannula. Routine use of a third arm for traction/counter-traction was highlighted; Maryland forceps were described as sharp but can become sticky after cautery, requiring cleaning
10.1002/lary.30882	Stefan et al.	Single-Port Robotic Removal of a Submucosal Foreign Body in the Distal Hypopharynx	N/A (foreign body removal; no tumor)	Operative set-up: Feyh–Kastenbauer retractor with hypopharyngeal blade; single-port system with monopolar scissors and Maryland dissector; assistant suction	None	N/A	Case complication: Immediate tongue oedema after extubation resolved with dexamethasone. Postoperative day 7 imaging showed a small hypopharyngeal leak; managed conservatively (nasogastric and gastrostomy tubes) and resolved by 4 weeks	Leak-related feeding: Required 4 weeks of enteral feeding via nasogastric and gastrostomy tubes until leak resolution	N/A	Single cannula reduced instrument clashes at the oral inlet; flexible three-dimensional high-definition endoscope improved visualization/access (including distal hypopharynx); articulating endoscope and arms improved dissection, retraction, haemostasis, suturing versus Xi or microlaryngeal instruments
10.1016/j.oor.2024.100547	Gorelik et al.	Single port robotic surgery for pleomorphic adenomas of the parapharyngeal space	Benign parapharyngeal space tumors: Two prestyloid deep-lobe lesions; both pleomorphic adenomas. Sizes on magnetic resonance imaging: 2.9 × 5.3 × 4.9 cm (left) and 3.6 × 2.2 × 3.1 cm (right)	N/A	None	Extracapsular dissection with no capsule disruption in either case	No significant long-term nerve deficits or complications	Case 1: well healed and tolerating a full liquid diet at 6 months; no long-term nerve deficits. Case 2: disease free after revision surgery and adjuvant radiotherapy; no persistent cranial nerve deficits	Case 1: No recurrence at 6 months after surgery. Case 2: Local recurrence at 12 months (0.6 × 0.9 × 0.9 cm pleomorphic adenoma in PPS) treated with repeat SP TORS plus adjuvant radiotherapy; disease-free at 3-month post-treatment follow-up	Flexible endoscope provided strong visualization beyond direct line of sight; single cannula improved range of motion/instrument access; third arm enabled static retraction and traction–countertraction; no major haemorrhage or capsule violation reported with careful technique
10.1007/s11701-025-02699-2	Fang et al.	Evaluation of single-port TORS tongue base resection for obstructive sleep apnea: safety and patient outcomes	N/A (obstructive sleep apnoea; no tumor)	Docking 5.0 ± 2.0 min (range 2.0–12.0, median 4.0); console 74.0 ± 14.9 min; tongue base resection time 23.0 ± 10.0 min (range 10–39)	None	N/A	No perioperative haemorrhage requiring return to theatre; one minor delayed bleed. Tongue swelling and taste disturbance reported; no feeding tubes or postoperative breathing difficulty	Obstructive sleep apnoea protocol and symptom improvement: Standard diet progression from liquids to soft foods starting postoperative day 2; no feeding tubes. Pain decreased from 6.5 ± 2.3 (day 1) to 3.4 ± 2.3 (day 7); Epworth Sleepiness Scale improved 9.6 ± 3.0 → 3.9 ± 2.3 at 1 month; mean length of stay 7.3 ± 1.1 days	N/A	Docking 5.0 ± 2.0 min (range 2–12, median 4). Lateral mouth extension 9.2 ± 0.6 cm; vertical opening 3.5 ± 0.6 cm
10.1002/hed.28007	Jeong et al.	First Prospective Study on Single-Port Robotic Tongue Base Resection for Sleep Apnea	N/A (obstructive sleep apnoea; no tumor)	Total time median 116 min (interquartile range 92–140); docking median 29 min (interquartile range 24–34); console median 25 min (interquartile range 21–32); estimated blood loss median 18 mL (interquartile range 10–30); resected tissue volume median 7.0 mL (interquartile range 5–9)	None	N/A	N/A	Diet advanced from liquids to soft foods starting the day after surgery; MD Anderson Dysphagia Inventory global/composite scores showed no deterioration. Daytime sleepiness improved (Epworth Sleepiness Scale median 10 → 6, *p* < 0.001; Stanford Sleepiness Scale median 4 → 2, *p* = 0.002) and Berlin questionnaire risk category improved (high risk 88% → 40%, *p* = 0.001)	N/A	Docking time median 29 min (interquartile range 24–34); console time median 25 min (interquartile range 21–32)
10.1007/978-3-031-96837-2_3	Chen et al.	Single-Port Robotic Free Flap Inset Following Transoral Robotic Surgery	Oropharyngeal squamous cell carcinoma requiring resection and free-flap reconstruction: Base of tongue/oropharyngeal wall reported; exact tumor stage not specified; included salvage context (prior partial glossectomy and chemoradiotherapy in at least one patient)	Resection + reconstruction (single port free-flap inset): Flaps: 1 radial forearm free flap and 2 anterolateral thigh flaps. Mean total operative time 573 min. Ischemia time (including robotic inset) decreased with experience: 198, 146, and 124 min (cases 1–3)	None	All resection margins were negative	No flap losses or donor-site complications. One haematoma between native tongue and flap required emergent return to theatre for ligation of a small bleeder, with no subsequent flap or wound-healing issues	N/A	N/A	N/A

Qualitative system-performance findings were broadly consistent across studies. Authors frequently reported reduced external arm collisions with the single-port architecture, although internal instrument conflicts, including controllable “chopstick” collisions, were still described in some cases. Several studies emphasized the importance of arm positioning, camera flexibility, retractor choice, and assistant workflow. No study reported intraoperative conversion to multiport or open surgery.

### Overall complications

Bleeding was the most consistently reported surgical complication, and patient-level event rates were prioritized where available. Park et al. reported one postoperative tonsillar bleed requiring treatment under general anesthesia, with a mean blood loss of 126.5 mL and no transfusions [16]. In Holsinger et al., mean estimated blood loss was 15.4 ± 23.9 mL with no transfusions, while grade III–IV postoperative haemorrhage, classified according to the Clavien–Dindo system, requiring return to theatre occurred in 2/47 patients (4%) [17]. Chan et al. reported an estimated blood loss of 39.2 mL (range 5.0–100.0) and no serious robot-attributed adverse events within 30 days; complications occurred in 8/21 patients and were mostly low-grade [18]. Jeong et al. reported a median estimated blood loss of 18.0 mL, and Fang et al. reported one minor delayed bleed without return-to-theatre haemorrhage [25, 26].

In larger comparative cohorts, Van Abel et al. reported haemorrhage in 4/78 patients (5.1%), while Costantino et al. reported major bleeding requiring haemostasis under general anesthesia in 6/209 patients (2.9%; single-port 3.7% vs. multiport 2.7%, *p* = 0.90) [9, 22]. Other infrequent complications included laryngeal swelling requiring PEG and pharyngeal fistula in Sampieri et al. (4/112 patients, 3.6%, with Clavien–Dindo > II), transient tongue oedema and a small hypopharyngeal leak resolving by 4 weeks in Stefan et al., and one flap-site haematoma without flap loss in Chen et al. [20, 23, 27]. All reported complications are listed in Table 2.

### Functional outcomes

Functional outcomes were reported heterogeneously and included oral intake, feeding tube dependence, tracheostomy dependence, length of stay, dysphagia scores, and sleep-related outcomes. Overall, the available data suggested favorable early functional recovery in selected patients, particularly with respect to oral intake. In Holsinger et al., 45/47 patients were eating by mouth without a percutaneous endoscopic gastrostomy tube within 30 days [17]. In Park et al., hypopharyngeal cancer patients resumed oral intake within 11 days, and temporary tracheostomies were decannulated within 28 days [16]. Longer-term dependence was uncommon in Costantino et al., with 6-month tracheostomy dependence in 3/209 patients and feeding tube dependence in 1/209 patients [22].

Swallowing and quality-of-life outcomes were less consistently reported. Chan et al. observed early worsening of dysphagia-related scores at 2 weeks with improvement toward baseline by 30 days [18]. In OSA cohorts, diet advancement was generally feasible without feeding tubes, and subjective sleepiness improved after surgery. Because outcome instruments, follow-up intervals, and reporting formats differed substantially, these functional findings should be interpreted descriptively rather than as definitive evidence of functional benefit.

### Oncological outcomes

Oncological outcome reporting was limited and focused mainly on margin status, adjuvant therapy, and short-term recurrence. Several cohorts reported high negative-margin rates, including Holsinger et al. with negative margins in 39/40 malignant cases, Chan et al. with negative margins in all listed malignant resections, and Van Abel et al. with 100% negative margins in chart-reviewed SP cases [9, 17, 18]. Park et al. also reported predominantly negative margins across laryngeal, hypopharyngeal, and oral cavity/oropharyngeal sites [16].

However, margin outcomes were not uniformly favorable across all studies. Sampieri et al. reported negative margins in 59/112 patients and positive margins in 53/112 patients, while Costantino et al. reported SP margins as negative in 96 patients, close in 13 patients, and positive in 27 patients [20, 22]. These differences likely reflect variation in tumor subsite, stage, exposure, pathological margin definitions, and case selection. Long-term oncological endpoints, including locoregional control, disease-free survival, and overall survival, were sparsely reported; therefore, no firm conclusions regarding durable oncological control can be drawn from the current evidence.

### Meta-analysis

**Fig. 3:**
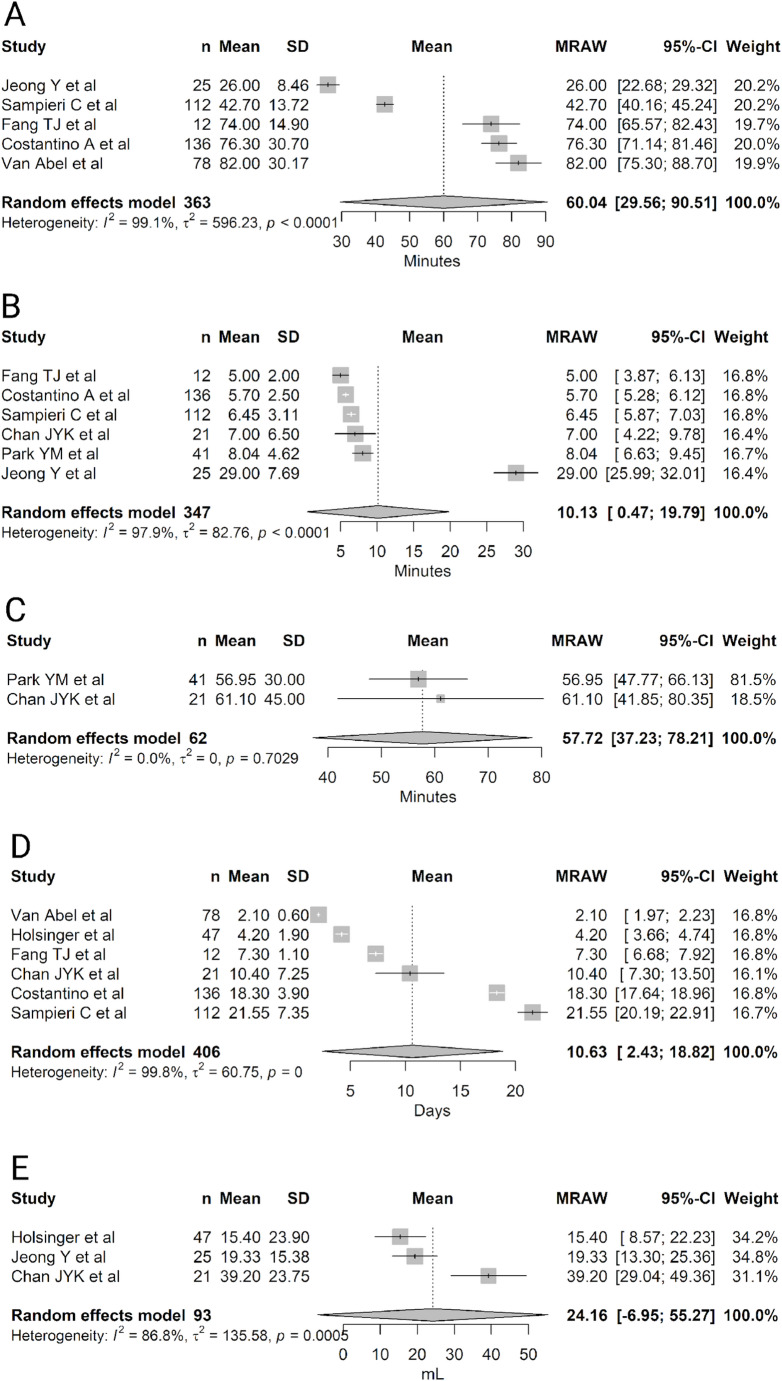
Forest plots of numerical outcome measurements (**A**: Console time, **B**: Docking time, **C**: Operative time, **D**: Length of stay, E: Estimated blood loss). Given the early-stage evidence base and substantial heterogeneity in several outcomes, pooled estimates should be interpreted as exploratory rather than conclusive

In the random-effects meta-analysis, pooled console time was 60.04 min (95% CI 29.56–90.51) with very high heterogeneity (I² = 99.1%, *p* < 0.0001) (Fig. 3A). Pooled docking time was 10.13 min (95% CI 0.47–19.79), with substantial heterogeneity (I² = 97.9%, *p* < 0.0001) (Fig. 3B). Pooled operative time was 57.72 min (95% CI 37.23–78.21) with no observed heterogeneity (I² = 0.0%, *p* = 0.70) (Fig. 3C). Pooled length of stay was 10.63 days (95% CI 2.43–18.82) with marked heterogeneity (I² = 99.8%, *p* < 0.0001) (Fig. 3D). Finally, pooled estimated blood loss was 24.16 mL (95% CI − 6.95 to 55.27) and high heterogeneity (I² = 86.8%, *p* = 0.0005) (Fig. 3E). Given the early-stage nature of the available SP-TORS evidence, the limited number of contributing studies, and the substantial heterogeneity observed for several outcomes, these pooled findings should be interpreted as exploratory and hypothesis-generating rather than conclusive or clinically definitive estimates.

## Discussion

This systematic review and meta-analysis included 14 studies evaluating da Vinci SP-TORS across oncologic, sleep-surgery, reconstructive, benign, and preclinical settings. The available evidence supports the technical feasibility of SP-TORS in selected patients, with no reported intraoperative conversions to multiport or open surgery and generally low estimated blood loss. However, perioperative estimates showed substantial between-study heterogeneity, while functional and oncological outcomes were inconsistently reported and often limited by short follow-up. Therefore, the current literature should be interpreted primarily as early clinical experience rather than definitive evidence of superiority or comparative effectiveness over established multiport systems.

**Fig. 4:**
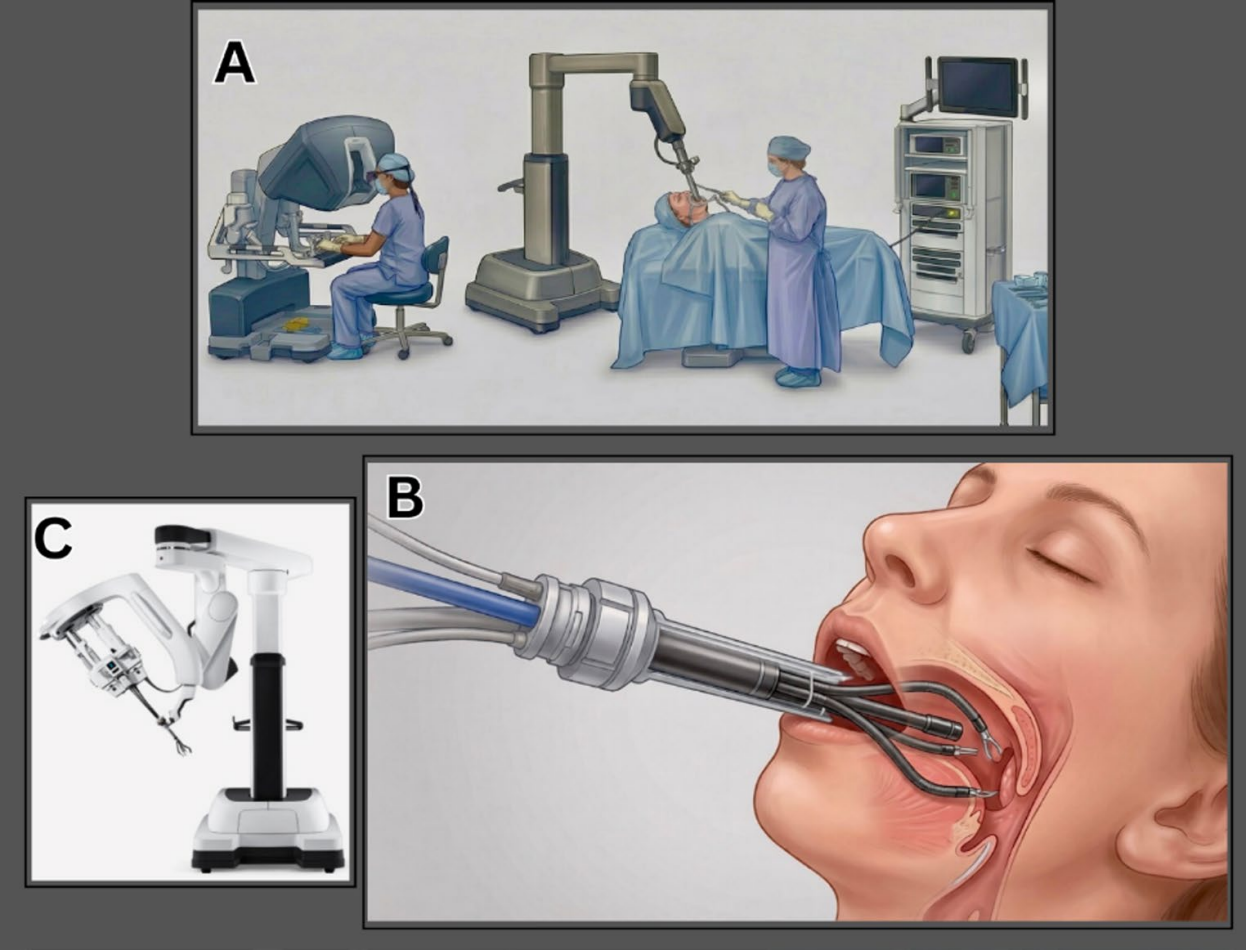
Operational Configuration and Clinical Application of the da Vinci Single-Port (SP) System in Transoral Robotic Surgery (TORS). The composite figure illustrates the SP platform’s workflow and anatomical access: (**A**) Schematic Illustration of the Intraoperative Environment: Layout of the surgical suite showing the surgeon at the ergonomic console, the patient cart, and the vision cart for 3D visualization. (**B**) Schematic Illustration of the Transoral Approach: Endoluminal access via a single 25-mm cannula, demonstrating the deployment of articulated instruments for triangulated access to the oropharynx and larynx. (**C**) Photographic Representation of the SP Platform: High-resolution image of the robotic unit highlighting the unified arm design and multi-jointed instruments engineered for restricted surgical corridors

These findings are consistent with earlier TORS literature showing that robotic transoral approaches can provide minimally invasive access to selected oropharyngeal, tongue-base, laryngeal, and hypopharyngeal lesions (including de-intensification paradigms in HPV-associated OPSCC) [13–18]. Beyond the da Vinci ecosystem, feasibility and early clinical outcomes have also been reported using alternative transoral robotic platforms (e.g., flexible robotic systems), highlighting that the key limiting factor is often transoral access and exposure rather than the brand-specific robot itself [10]. The absence of conversions in the SP-TORS literature likely reflects both careful patient selection at experienced centers and platform-specific advantages (single cannula architecture, flexible camera and multi-articulated instruments) that reduce arm collisions in narrow corridors (Fig. 4) [7, 16, 17]. Earlier multiport TORS experience has shown that inadequate exposure can preclude transoral robotic resection in a subset of patients and that occasional conversion to open surgery may occur [28]. Clinically, these findings support SP-TORS as a feasible option in selected patients, but they should be interpreted in the context of early adoption, where early experience and publication bias tend to favor technically successful cases.

We found pooled estimates indicating efficient operative performance (operative time ~ 58 min; docking time ~ 10 min; console time ~ 60 min), yet substantial between-study heterogeneity for several time outcomes. These pooled values are directionally consistent with institutional comparisons reporting shorter setup/docking times and fewer collisions with SP versus legacy platforms, but reported times vary widely across subsites (oropharynx vs. larynx/hypopharynx), indication (oncologic vs. OSA vs. reconstruction), and differences in what authors label as “docking,” “setup,” and “console” time [7, 9, 16, 20, 22]. This heterogeneity likely reflects, at least in part, differences in outcome definitions, procedure types, subsites, exposure strategies, and institutional learning curves. However, because the primary studies did not uniformly define the components of docking, setup, console, and operative time, these factors should be interpreted as likely contributors rather than formally proven determinants of heterogeneity.

Learning-curve signals reported in the SP series mirror the broader TORS literature, in which experience and standardized pathways strongly influence efficiency [9, 29, 30]. From a clinical operations standpoint, the implication is that centers introducing SP-TORS should expect initial variability and should prospectively define time endpoints, implement standardized docking/checklists, and report times by subsite and procedure type to enable meaningful benchmarking and future comparative trials.

In our meta-analysis, the pooled estimated blood loss was low (~ 24 mL), consistent with the minimally invasive transoral approach. Nevertheless, bleeding was the most frequently reported postoperative complication across the included studies, with some cohorts reporting episodes that required return to the operating room. This pattern is consistent with the broader TORS evidence base identifying postoperative hemorrhage as the most common serious complication, with reported incidence in systematic reviews commonly in the mid-single digits and with rare severe events [31–34]. In addition to immediate morbidity, postoperative hemorrhage can have downstream consequences, including delayed recovery and potential disruption of adjuvant treatment timelines, which is particularly relevant in OPSCC pathways where coordination of postoperative therapy is time-sensitive [35]. Strategies such as prophylactic transcervical arterial ligation have been examined in both meta-analyses and cohort studies and may reduce the severity of major hemorrhage, though effects on overall bleeding incidence vary [32, 36, 37]. SP-TORS may influence hemorrhage risk in two countervailing ways: improved access/visualization could facilitate meticulous hemostasis, while deeper subsites (hypopharynx/larynx) becoming more accessible might increase exposure to vessels and challenging angles, particularly during early adoption. Clinically, these findings support the use of predefined hemorrhage-prevention pathways rather than ad hoc management. Such pathways may include preoperative assessment of anticoagulation and bleeding risk, consideration of tumor subsite and extent of resection, standardized intraoperative hemostasis, selective prophylactic arterial ligation in high-risk oropharyngeal cases, structured postoperative monitoring, patient education regarding delayed hemorrhage, and clear emergency protocols for rapid evaluation and return to theatre when needed.

The pooled LOS was approximately 10.6 days but exhibited substantial heterogeneity, with individual studies ranging from short-stay pathways to prolonged admissions. This divergence likely reflects substantial differences in healthcare systems, discharge criteria, perioperative tracheostomy practices, feeding protocols, and indication mix (e.g., hypopharyngeal cancer resections with tracheostomy vs. straightforward oropharyngeal resections). Similar variability has been reported historically in multiport TORS series, where LOS is tightly linked to institutional pathways and complication profiles rather than technology alone [29, 38, 39]. Comparative SP vs. multiport cohorts often show modest reductions in LOS, but interpretation is complicated by contemporaneous changes in enhanced recovery practices and selection (earlier-stage tumors, improved anesthesia, refined swallowing pathways) [9, 20, 22]. Clinically, LOS should not be treated as an intrinsic advantage of SP-TORS; rather, it is an outcome that can be optimized through standardized post-TORS pathways, early swallow evaluation, proactive airway planning, and consistent criteria for tracheostomy and feeding tube use. Future studies should stratify LOS by subsite and pathway variables and consider reporting “time to oral intake” alongside standardized discharge-readiness criteria, such as stable airway status, absence of active bleeding concerns, adequate pain control, ability to maintain hydration and nutrition orally or through a defined enteral-feeding plan, completed swallowing assessment when indicated, and clear postoperative emergency instructions.

Across included cohorts, functional recovery signals, such as early return to oral intake, low long-term feeding tube dependence, and low rates of long-term tracheostomy dependence, were generally favorable, but endpoints were inconsistently defined and measured. Importantly, gastrostomy dependence after transoral surgery has been linked to factors such as baseline swallowing function, extent of resection, and (especially) adjuvant therapy intensity; therefore, inter-study differences in G-tube rates likely reflect case mix and adjuvant selection as much as surgical platform [32]. This mirrors the broader field: swallowing and quality-of-life outcomes after transoral surgery are highly dependent on adjuvant therapy intensity, baseline function, tumor subsite, and measurement instrument selection [39–42]. Randomized evidence comparing TORS-based strategies with definitive radiotherapy in OPSCC shows nuanced trade-offs across quality-of-life domains and underscores that surgical approaches do not automatically translate into superior swallowing outcomes without careful deintensification of adjuvant therapy [40, 43]. This is consistent with broader comparative syntheses suggesting that functional outcomes vary by endpoint and follow-up window and may not uniformly favor surgical or nonsurgical strategies, particularly after accounting for adjuvant therapy intensity [44]. In SP-TORS series, early swallowing recovery may be influenced by reduced external arm crowding and potentially improved precision in confined anatomy, but these hypotheses remain unproven because few studies incorporate rigorous, longitudinal, validated swallowing instruments with prespecified follow-up windows. Clinically, SP-TORS should be positioned as a tool that may facilitate function-preserving surgery; however, patient counseling must emphasize that functional outcomes are largely driven by tumor biology, extent of resection, and adjuvant treatment decisions. Trials and registries should adopt standardized endpoints (e.g., MDADI, G-tube dependence at 6/12 months, objective swallow studies) to allow fair comparisons across platforms.

Oncologic endpoints in the SP-TORS literature were variably reported, yet several studies demonstrated high negative margin rates, while others reported substantial rates of close/positive margins. This variability is not unique to SP-TORS: margin definitions (inked margin vs. distance thresholds), specimen orientation, and pathologic processing differ widely across institutions, which complicates comparisons even in multiport TORS cohorts [38, 45, 46]. Comparative SP vs. multiport studies in OPSCC and laryngo-hypopharyngeal disease have reported similar or favorable margin outcomes with SP in some settings, but the direction and magnitude of effect are inconsistent and may be confounded by selection (smaller tumors or better exposure chosen for SP early in adoption) [9, 20, 22]. A plausible mechanistic hypothesis is that SP’s flexible camera and internal instrument articulation may improve access to deep or curved anatomy, potentially enhancing en bloc resection and visualization of margin planes, yet limitations such as reduced instrument independence or “chopstick” collisions could counteract this advantage in challenging exposures. Clinically, the current evidence supports SP-TORS as oncologically feasible, but it remains premature to infer oncologic superiority or equivalence based solely on margins. Consistent reporting of pT/pN stage, margin definitions, adjuvant therapy, recurrence patterns, and follow-up duration is essential to determine durable cancer control.

Our included studies also demonstrate the expanding use of SP-TORS beyond oncologic resections, including OSA-related tongue base surgery, foreign body removal, parapharyngeal space tumors, and reconstructive surgery (e.g., flap inset). Early reports also describe SP-enabled transoral resections in challenging anatomic subsites such as supraglottic, supporting the concept that internal instrument articulation and camera flexibility may broaden candidacy for minimally invasive transoral approaches in carefully selected patients [6]. This trajectory resembles earlier multiport TORS diffusion from OPSCC into sleep surgery and benign pathology [29, 42]. Emerging SP-TORS tongue base data report improvements in subjective sleepiness metrics and feasibility, but robust comparative evidence against established approaches (e.g., coblation, non-robotic transoral techniques, multilevel surgery protocols) remains limited [26]. For reconstruction, SP-assisted inset is conceptually attractive because deep oropharyngeal access can be a limiting step in free-flap reconstruction, but this use-case requires dedicated evaluation of flap outcomes, operative time impact, and resource utilization. Clinically, the key implication is that SP-TORS “versatility” should not be interpreted as universal benefit; rather, each indication needs procedure-specific endpoints (AHI and objective sleep outcomes for OSA; margin and recurrence outcomes for oncology; flap viability and functional recovery for reconstruction) before routine adoption can be recommended.

Patient selection remains a central consideration when interpreting comparisons between SP and multiport TORS. SP-TORS may be particularly attractive in patients with adequate mouth opening but narrow or deep working corridors in which external arm collisions limit multiport access, including selected tongue-base, supraglottic, hypopharyngeal, parapharyngeal, and reconstructive applications. In contrast, multiport systems may remain suitable for cases with straightforward oropharyngeal exposure, procedures requiring wider instrument separation, or centers where multiport expertise and infrastructure are already well established. Importantly, early SP-TORS cohorts may overrepresent patients with favorable exposure, smaller tumors, or treatment at highly experienced robotic centers, which limits direct inference regarding platform superiority. Future comparative studies should therefore report selection criteria in detail, including interincisal distance, exposure quality, tumor subsite and size, reconstruction requirements, prior treatment, surgeon experience, and reasons for choosing SP versus multiport access.

A major unresolved question is whether SP platforms deliver clinically meaningful advantages over multiport TORS sufficient to justify acquisition and maintenance costs. Comparative clinical studies suggest potential workflow advantages (reduced collisions, potentially faster docking) but do not yet provide consistent evidence for superior clinical outcomes [7, 9, 20, 22]. Cost-effectiveness studies in transoral surgery have historically found that economic conclusions are highly sensitive to adjuvant therapy rates, revision surgery, and local cost structures; some analyses favor non-robotic transoral approaches, while others find TORS economically competitive depending on assumptions [47, 48]. Because SP adoption is often accompanied by contemporaneous improvements in pathways and shifts in case mix, isolating the platform’s incremental value is difficult. Since specific financial data were not extracted in this meta-analysis, the discussion regarding cost-effectiveness remains theoretical based on broader literature rather than derived directly from our pooled results. Clinically, centers should evaluate SP-TORS implementation using institution-specific health economic metrics (OR time, instrument utilization, length of stay drivers, complication costs, readmissions) and prioritize outcomes-based justification rather than purely technical enthusiasm.

The evidence base remains dominated by retrospective cohorts and early-phase experiences, and our pooled analyses underscore that inconsistent definitions and heterogeneous indications substantially limit inference. Accordingly, the findings of the present review should be interpreted as exploratory and hypothesis-generating rather than definitive. At present, the available data primarily support the technical feasibility and early clinical applicability of SP-TORS across selected settings, but they do not establish superiority in operative performance, functional outcomes, oncologic control, cost-effectiveness, or comparative effectiveness versus established surgical approaches. For surgical innovations, the IDEAL framework provides a practical roadmap for progression from development and exploration into robust assessment, emphasizing prospective data capture, transparency in reporting modifications, and ultimately randomized or well-controlled comparative studies where feasible [30]. In the case of oropharyngeal cancer, ongoing de-escalation efforts (e.g., risk-stratified adjuvant strategies) underscore that platform comparisons should be embedded within modern oncologic pathways rather than historical controls [40, 41, 49, 50]. Additionally, robotics might be supported by the implementation of artificial intelligence systems, telesurgery capabilities, and ethical frameworks in the future [51, 52]. Moreover, safety signals in deintensification trials highlight the importance of careful trial design, credentialing, and monitoring when evaluating surgical strategies [53]. Going forward, the most informative SP-TORS studies will be those that prospectively define endpoints (bleeding severity grading, standardized margin definitions, validated swallow measures, cost and resource outcomes), stratify by subsite and indication, and incorporate adequate follow-up to assess durable oncologic control and long-term function.

## Limitations

Several limitations should be considered when interpreting these findings. First, the evidence base was small (14 studies, 479 patients) and dominated by nonrandomized designs, including retrospective cohorts, case series/reports, and cadaveric feasibility studies, which increase susceptibility to selection bias and confounding, and limit external validity. Furthermore, the inclusion of case reports and small case series alongside larger cohorts introduces potential bias and limits the precision of pooled estimates. In addition, several pooled estimates were based on very few studies, particularly for operative time and estimated blood loss, limiting precision and further complicating interpretation of between-study variability. Moreover, the Google Scholar search was limited to the first 25 result pages, which may have introduced selection bias by excluding lower-ranked records; however, this limit was predefined and documented to improve reproducibility. Second, indications and case-mix were heterogeneous, including oncologic and non-oncologic applications, variable subsites and stages, as well as reconstructive, sleep surgery, and preclinical cadaveric cohorts. This clinical and methodological heterogeneity limits the ability to draw indication-specific conclusions and reduces the clinical interpretability of pooled estimates. Although oncologic outcomes were described separately where available, the scarcity and inconsistency of indication-specific data precluded a meaningful stratified quantitative analysis. Accordingly, the pooled findings should be interpreted as exploratory and hypothesis-generating rather than definitive. Outcomes were also inconsistently defined and reported, including variable docking-time definitions, different swallowing instruments and follow-up windows, and incomplete oncologic endpoints, contributing to the high statistical heterogeneity observed in the meta-analysis, with some I² values reaching approximately 99%. Third, comparative studies were often based on historical controls or practice-era differences (changes in perioperative pathways, tracheostomy practices, and discharge criteria), making it difficult to attribute observed differences solely to the single-port platform. Fourth, oncologic follow-up was generally short and recurrence, survival, and long-term functional outcomes were sparsely reported, limiting conclusions about durable cancer control and late toxicity. Finally, publication and reporting bias cannot be excluded, particularly given early adoption settings and the likelihood that favorable feasibility experiences are preferentially published, while cost-effectiveness and resource implications were rarely evaluated.

## Conclusion

Single-port transoral robotic surgery is feasible across a broad spectrum of head and neck indications, with predominantly oncologic applications and expanding adjunctive uses. Pooled estimates suggest efficient operative performance and generally low blood loss, with acceptable complication profiles and encouraging early functional recovery in reported cohorts. However, very high between-study heterogeneity and inconsistent outcome definitions limit the certainty of pooled perioperative estimates. Oncologic endpoints remain variably reported, with limited long-term follow-up despite generally high negative-margin rates in several series. Prospective comparative studies with standardized functional, oncologic, and cost-effectiveness outcomes are needed to define the clinical value of single-port platforms relative to multiport systems.

## Electronic Supplementary Material

Below is the link to the electronic supplementary material.

Supplementary Material 1

## Data Availability

No new data were generated or analyzed in this narrative review. All information is derived from previously published sources cited in the manuscript.
