# Supplementary material for: Transoral robotic surgery using the da Vinci single-port system: current evidence and clinical indications – a systematic review and meta-analysis
Source: J Robot Surg. 2026 Jun 12;20(1):586. doi: 10.1007/s11701-026-03540-0 (PMC13260184; doi:10.1007/s11701-026-03540-0)
Supplement: Supplementary file 1 — Supplementary Material 1 [file 11701_2026_3540_MOESM1_ESM.docx]

**Supplementary Information**

**Supplementary Digital Content 1.** Full search strategy for each database included

| **Database** | **Search String** | **Results** |
| --- | --- | --- |
| PubMed/MEDLINE | (("Transoral Robotic Surgery"[tiab] OR TORS[tiab] OR "transoral robot*"[tiab]) AND ("single port"[tiab] OR "single-port"[tiab] OR "single incision"[tiab] OR "single-incision"[tiab] OR SP[tiab] OR "da Vinci SP"[tiab] OR "single port system"[tiab] OR "single-port system"[tiab])) | 61 |
| EMBASE | ('transoral robotic surgery' OR tors OR transoral NEXT/1 robot*) AND ('single port' OR 'single-port' OR 'single incision' OR 'single-incision' OR 'da vinci sp' OR 'sp system' OR 'single port system' OR 'single-port system') | 365 |
| Google Scholar (First 25 pages) | ("transoral robotic surgery" OR TORS) ("single port" OR single-port OR "da Vinci SP" OR "SP system") | 250 |
| Cochrane | (TORS OR "transoral robotic surgery" OR transoral NEAR/1 robot*) AND ("single port" OR single-port OR "single incision" OR "da Vinci SP" OR "SP system" OR "single port system" OR "single-port system") | 3 |

**Supplementary Digital Content 2.** Level of Evidence (LOE) assessment of studies included

| **DOI** | **First author et al.** | **Study type** | **LOE-Score** |
| --- | --- | --- | --- |
| DOI: 10.1245/s10434-019-07802-0 | Park YM et al. | Retrospective case series | 4 |
| DOI: 10.1001/jamaoto.2019.2654 | Holsinger FC et al. | Phase 2, multicenter, prospective non-randomized clinical trial | 2 |
| DOI: 10.1002/hed.25676 | Orosco RK et al. | Preclinical cadaver feasibility/anatomic study | 5 |
| DOI: 10.1016/j.oraloncology.2019.05.018 | Chan JYK et al. | Prospective Phase II, IDEAL stage 2, single-arm clinical trial of safety and feasibility | 2 |
| DOI: 10.1002/hed.26143 | Van Abel KM et al | Retrospective cohort with historical Si comparison | 3 |
| DOI: 10.1002/hed.26794 | Mendelsohn AH et al. | Case report | 4 |
| DOI: 10.1002/ohn.287 | Sampieri C et al. | Retrospective cohort study with propensity score–matched comparison of SP vs multiport (Si/Xi) TORS | 3 |
| DOI: 10.1002/rcs.2510 | San Juan JD et al. | Preclinical feasibility and usability study in a laboratory setting using human cadaver specimens and trainee surveys | 5 |
| DOI: 10.1016/j.oraloncology.2023.106629 | Costantino A et al. | Single-center retrospective cohort study comparing da Vinci SP vs Xi in OPSCC patients treated with TORS after neoadjuvant chemotherapy | 3 |
| DOI: 10.1002/lary.30882 | Stefan AJ et al. | Case report | 4 |
| DOI: 10.1016/j.oor.2024.100547 | Gorelik D et al. | Case series | 4 |
| DOI: 10.1007/s11701-025-02699-2 | Fang TJ et al. | Non-randomized, single-arm phase II observational trial | 2 |
| DOI: 10.1002/hed.28007 | Jeong Y et al. | Prospective, single-center, single-surgeon clinical investigation | 3 |
| DOI: 10.1007/978-3-031-96837-2_3 | Chen B et al. | Case series | 4 |

**Supplementary Digital Content 3.** Newcastle-Ottawa Scale (NOS) assessment of studies included

| **DOI** | **First author et al.** | **Study type** | **NOS-Score** |
| --- | --- | --- | --- |
| DOI: 10.1001/jamaoto.2019.2654 | Holsinger FC et al. | Phase 2, multicenter, prospective non-randomized clinical trial | 6 |
| DOI: 10.1016/j.oraloncology.2019.05.018 | Chan JYK et al. | Prospective Phase II, IDEAL stage 2, single-arm clinical trial of safety and feasibility | 6 |
| DOI: 10.1002/hed.26143 | Van Abel KM et al | Retrospective cohort with historical Si comparison | 4 |
| DOI: 10.1002/ohn.287 | Sampieri C et al. | Retrospective cohort study with propensity score–matched comparison of SP vs multiport (Si/Xi) TORS | 4 |
| DOI: 10.1016/j.oraloncology.2023.106629 | Costantino A et al. | Single-center retrospective cohort study comparing da Vinci SP vs Xi in OPSCC patients treated with TORS after neoadjuvant chemotherapy | 4 |
| DOI: 10.1007/s11701-025-02699-2 | Fang TJ et al. | Non-randomized, single-arm phase II observational trial | 6 |
| DOI: 10.1002/hed.28007 | Jeong Y et al. | Prospective, single-center, single-surgeon clinical investigation | 5 |

**Supplementary Digital Content 4.** Joanna Briggs Institute critical appraisal tool assessment for case reports and case series of studies included

| **JBI Item** | **Park YM et al.** | **Gorelik D et al.** | **Chen B et al.** | **Mendelsohn AH et al.** | **Stefan AJ et al.** |
| --- | --- | --- | --- | --- | --- |
| *Study Type* | *Case Series* | *Case Series* | *Case Series* | *Case Report* | *Case Report* |
| 1. Clear inclusion criteria / Demographics clearly described? | Yes | Unclear | Unclear | Yes | Yes |
| 2. Condition measured standardly / History as timeline? | Unclear | Unclear | Unclear | Unclear | Yes |
| 3. Valid identification methods / Clinical condition at presentation? | Yes | Yes | Unclear | Yes | Yes |
| 4. Consecutive inclusion / Diagnostic tests described? | No | Unclear | No | Yes | Yes |
| 5. Complete inclusion / Intervention clearly described? | Unclear | Unclear | Unclear | Yes | Yes |
| 6. Demographics reported / Post-intervention condition? | Yes | Unclear | Unclear | Unclear | Unclear |
| 7. Clinical information reported / Adverse events described? | Yes | Unclear | Unclear | Unclear | Unclear |
| 8. Outcomes clearly reported / Takeaway lessons? | Yes | Unclear | Unclear | Yes | Yes |
| 9. Site/clinic demographics reported? | Unclear | Unclear | Unclear | — | — |
| 10. Statistical analysis appropriate? | Unclear | Unclear | N/A | — | — |
| Overall | Low–Moderate | Low | Low | Low–Moderate | Low–Moderate |
